# Computational simulations of the effects of gravity on lymphatic transport

**DOI:** 10.1093/pnasnexus/pgac237

**Published:** 2022-10-18

**Authors:** Huabing Li, Huajian Wei, Timothy P Padera, James W Baish, Lance L Munn

**Affiliations:** Department of Radiation Oncology, Massachusetts General Hospital and Harvard Medical School, Boston, MA 02114, USA; Department of Material Science and Technology, Guilin University of Electronic Technology, Guilin 541004, China; Department of Material Science and Technology, Guilin University of Electronic Technology, Guilin 541004, China; Department of Radiation Oncology, Massachusetts General Hospital and Harvard Medical School, Boston, MA 02114, USA; Biomedical Engineering, Bucknell University, Lewisburg, PA 17837, USA; Department of Radiation Oncology, Massachusetts General Hospital and Harvard Medical School, Boston, MA 02114, USA

**Keywords:** lymphatic vessel pumping, edema, gravity, valves, computational model

## Abstract

Physical forces, including mechanical stretch, fluid pressure, and shear forces alter lymphatic vessel contractions and lymph flow. Gravitational forces can affect these forces, resulting in altered lymphatic transport, but the mechanisms involved have not been studied in detail. Here, we combine a lattice Boltzmann-based fluid dynamics computational model with known lymphatic mechanobiological mechanisms to investigate the movement of fluid through a lymphatic vessel under the effects of gravity that may either oppose or assist flow. Regularly spaced, mechanical bi-leaflet valves in the vessel enforce net positive flow as the vessel walls contract autonomously in response to calcium and nitric oxide (NO) levels regulated by vessel stretch and shear stress levels. We find that large gravitational forces opposing flow can stall the contractions, leading to no net flow, but transient mechanical perturbations can re-establish pumping. In the case of gravity strongly assisting flow, the contractions also cease due to high shear stress and NO production, which dilates the vessel to allow gravity-driven flow. In the intermediate range of oppositional gravity forces, the vessel actively contracts to offset nominal gravity levels or to modestly assist the favorable hydrostatic pressure gradients.

Significance Statement The mechanisms that result in lymphatic insufficiency and peripheral lymphedema are not completely understood, but can be exacerbated by gravitational forces, especially in the lower limbs. To better understand the interplay between tissue fluid pressure, lymphatic contractions, and gravitational forces, we developed a mathematical model that includes the relevant mechanobiological mechanisms. The simulations show that lymph transport is most efficient when the limb drainage is assisted by gravitational forces and decreases when the flow direction is opposed by gravity. The results also suggest that lymphatic contractions can be stalled by excessive body forces and re-activated by tissue-induced mechanical perturbations.

## Introduction

Lymphatic transport of fluid and cells is important for fluid homeostasis and the immune response. Collecting lymphatic vessels can actively pump fluid, but lymphatic pathologies can result in inefficient transport and accumulation of fluid (lymphedema) (1–4). It is well-established that lymph transport and fluid homeostasis are affected by gravitational forces and limb orientation (5, 6). Lymphatic insufficiency is exacerbated if the direction of fluid evacuation is opposed by gravity (7, 8).

The fact that lymphedema often occurs in dependent limbs highlights the importance of gravity-imposed pressure on fluid homeostasis and lymph drainage (9, 10). An early study by Olszewski and Engeset addressed the question of whether evacuation of fluid from the lower limbs is driven by skeletal muscle movement or intrinsic lymphatic vessel contractions. By carefully measuring vascular pressures in various conditions (standing, supine, with and without flexing the foot), they found no differences in lumen pressure within the lymphatic vessels between standing and recumbent conditions, but saw increases in contraction frequency when standing or in response to external massaging of the foot. Interestingly, they observed long periods with no contractions of the vessels, but the contractions could be initiated by injecting fluid (to increase pressure) or massaging the area adjacent to the vessel (11).

Other studies have observed similar changes in contraction frequency between upright and recumbent positions. Holm-Weber et al. reported a more than doubling in frequency 3 minutes after standing from a recumbent position (12). This higher frequency persisted for up to 6 minutes after the subject returned to a lying position. In this study, there was also an increase in lymph pressure of 9 mmHg when standing relative to lying down. As a result of this and other studies, a common therapeutic recommendation for peripheral edema is to elevate the affected limb(s) so gravity can assist, rather than oppose, drainage. However, benefit may be lost unless the elevation is maintained. The fluid pressure tends to increase quickly when the limb is returned to a dependent position (13).

These considerations are also relevant to prolonged space flight, where an absence of gravity induces many changes in physiology (14, 15). It has been suggested that these changes are due to cytoskeletal filament rearrangement, endothelial dysfunction, and muscular atrophy, which affect vascular function and fluid homeostasis (16). Evidence from mouse experiments in microgravity support the idea that lymph flow is affected by gravitational forces. After returning from a 13 day space shuttle mission, mice had significantly altered systemic distributions of T cells (17).

The relationship between cerebrospinal fluid (CSF) pressure and gravitational forces is of primary importance in prolonged space flight. CSF accumulation occurs in a number of pathological conditions, and can result in spaceflight-associated neuro-ocular syndrome, a problem for long-duration spaceflight. Studies have shown that the fluid accumulation is due to the altered gravitational environment and involves changes in lymphatic contractility (18).

In vitro experiments can be leveraged to study lymphatic physiology under controlled conditions. By isolating and cannulating lymphatic vessels ex vivo, it is possible to examine the relationships between lumen pressure and contractility (19). These studies show that increasing pressure in the lymphatic lumen results in altered contraction frequency and output (20). Lymphatic muscle cells adapt to changes in transwall pressure with increased contractility to maintain homeostasis (21). However, studies that impose only a pressure within the vessel may not completely recapitulate the effects of gravitational forces, which operate both inside and outside the vessel wall, so have less impact on transwall forces. Although much clinical evidence and basic research shows correlations between lymphatic function and gravitational forces, there are still outstanding questions concerning how lymphatic function is affected by different body forces (18, 22–26).

Previous mathematical models have been developed to investigate lymphatic function and fluid homeostasis. Detailed models of lymphatic networks, complete with valves have been described (27), and valve mechanics have been examined in detail using explicit mechanics and fluid dynamics (28). Other models have examined the effects of gravity on CSF pressures (29) and the effects of microgravity on systemic fluid redistribution using whole-body compartment-based modeling (30). In general, these models examine transport without consideration of the mechanobiological mechanism that control lymphatic contractions.

Previously, we demonstrated how the dynamics of nitric oxide (NO) (produced by shear stress on lymphatic endothelial cells) and intracellular calcium fluxes in lymphatic muscle cells can establish feedback that controls lymphatic contractions (31–33). We have also considered the effects of gravity on the fluid balance in the lower leg at the scale of the entire limb using a simplified model of lymphatic pumping (57), but did not address the pumping dynamics of individual lymph vessels. Here, we analyze how gravitational force (i.e. limb position) affects lymphatic vessel contractions, tissue fluid pressure, and valve dynamics using a lattice Boltzmann mathematical model.

## Model summary

**Fig. 1.**
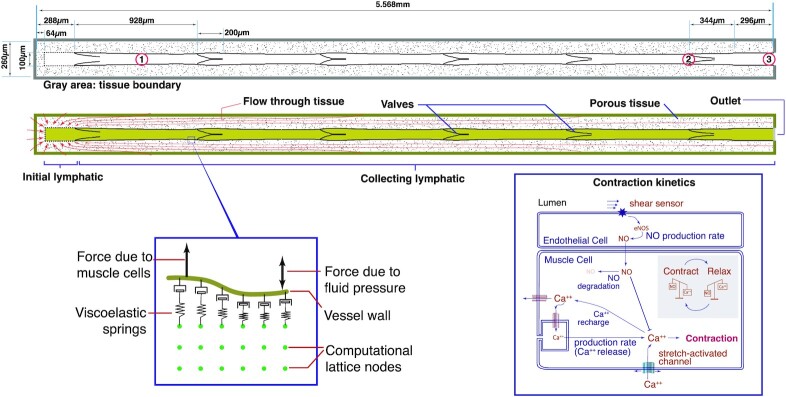
Schematic of computational domain. At left is the initial lymphatic vessel segment where fluid enters the vessel, bounded by the dashed line. Fluid enters this region from the surrounding tissue (red arrows). The initial lymphatic vessel segment is connected to the collecting lymphatic vessel segment, which contains a series of lymphangions (five are shown in this example). The extravascular tissue is modeled as a porous material by including solid islands (black dots), which hinder fluid flow in the lattice Boltzmann calculations. The gray boundary is the interface with the surrounding tissue; fluid freely enters at this boundary. Flow leaves the domain at the exit of the vessel (right). The three locations indicated by red circles are referenced in the results section. The vessel wall is anchored with viscoelastic springs and is affected by calcium-induced muscle cell contractions as well as fluid forces (detail at bottom left). The muscle cell forces are modeled using a simplified scheme of calcium dynamics (bottom right). Calcium enters the cytoplasm to induce contractions, and its activity is countered by NO produced by endothelial shear stress (35).

Our 2D model domain consists of an initial lymphatic vessel where fluid enters the vessel, several collecting lymphangions in series, and one outlet lymphangion (Fig. 1); each lymphangion is flanked by two valves (34). The vessel is embedded in a porous tissue space where fluid can move in response to pressure gradients. The outer domain boundary (gray line) represents the interface with the surrounding tissue. The outer domain boundary is sufficiently distant from the vessels so as to not affect wall motion. Similarly, the outer domain boundary is porous, allowing fluid to leave or enter the domain, thus fluid–wall interactions should not be affected by the boundary.

### Fluid dynamics

Fluid dynamics are simulated by the D2Q9 lattice Boltzmann method (LBM) (36). Interactions with the moving vessel wall and the valves are calculated according to the so-called “curved boundary condition” (37), and the hydrodynamic force is integrated using the stress-integration method (38). Fluid interaction with solid islands in the tissue, the inlet of the initial lymphangion, and the static region at the vessel outlet are treated with bounce-back boundary conditions (39). Similarly, the fluid exchanges momentum with the vessel wall and valves. To simulate gravity, a body force is applied to all the fluid in the domain, including the extravascular space. The direction is dictated by the vessel orientation.

### Tissue domain

Fluid moves into the domain from the surrounding tissue across the gray boundary in Fig 1. In the tissue domain region, pressure gradients drive the flow, which is generally oriented toward the initial lymphatic segment. To simulate flow in a porous media, the tissue domain includes randomly placed solid “islands” (black dots, Fig. 1); these comprise 5% of the tissue nodes outside the lymphatic vessel. In the LBM code, fluid packets that encounter these islands are reflected locally (implemented using a full bounce-back condition) (40), thus hindering convection.

### Initial lymphatic region

The initial lymphatic capillary is bounded by the dashed rectangle at the left of the domain (Fig. 1). The wall in this region is rigid, but contains a number of apertures that represent primary valves through which flow can pass. These apertures comprise 50% of the wall. At these locations, fluid freely enters if the pressure gradient is favorable; some fluid can also exit the region if the lumen pressure is higher than the tissue pressure.

In our simulations, a small amount of retrograde flow is necessary to close the first intraluminal valve. Experimental evidence suggests that the primary valves of initial lymphatic vessels normally do not allow leakage of injected tracer when the lymphatic pressure exceeds that of the tissue; however, during inflammation, backflow is possible (41). In addition, it is likely that initial and precollector lymphatics have elastic compliance that allows for some retrograde flow that is necessary to close the valve leaflets in the distal region of the network (22). Thus, our imposition of slightly leaky primary valves is reasonable as it does not alter the main model findings and compensates for the lack of compliance upstream from the first valve. To do this, we impose the leakage of the lymph out of the initial lymphatic vessels using a partial bounce-back condition so that during vessel contraction, some of the flow initially goes backward through the (slightly leaky) initial lymphatic vessels into the tissue. This small amount of retrograde flow from the first lymphangion is sufficient to close the first open intraluminal valve. Specifically, fluid entering the vessel passes freely through the gaps in the initial lymphatic shown in Fig. 1; however, when the pressure gradient favors back flow into the tissue, 85% of fluid is reflected and 15% is allowed to leak back into the tissue. This is imposed using a partial bounce-back boundary condition (40), and approximates the primary “flap” valves (42, 43) as well as upstream lymphatic compliance.

### Collecting lymphatic vessel

The initial lymphatic segment connects to the collecting lymphatic vessel, which consists of a number of lymphangions in series, separated by explicit mechanical valves. The vessel wall in this segment is flexible, and is anchored to the surrounding tissue with visco-elastic springs (33). Transwall fluid pressure gradients can displace the wall locally, implemented by calculating the momentum transfer between the fluid and wall nodes (33, 44). Conversely, the wall generates forces that can move the fluid. We do not explicitly model deformation of the tissue outside the vessel, but instead adjust the tissue and fluid node conditions in the LBM code: when the vessel wall expands into this tissue, we replace tissue nodes with fluid at those locations. Similarly, when the vessel wall recedes, fluid nodes are replaced by tissue nodes as appropriate (44). To anchor and stabilize the outlet region of the collecting vessel, we impose a 0.296 mm length of noncontracting vessel extending to the outlet boundary. The total vessel length is 5.6 mm for the 5-lymphangion vessel and 29.7 mm for the 31-lymphangion vessel.

At the vessel outlet, we impose a pressure boundary condition (45). To simulate the fluid dynamics of lymphangions and valves downstream, beyond the domain, we introduce a partial bounce-back boundary located  0.016 cm upstream from the vessel outlet. At this boundary, fluid can exit freely, but if the flow reverses, 85% will be reflected. When the last valve is completely closed, the bounce-back on this boundary increases to 100%. This simulates the effects of downstream valves, and is necessary to prevent instabilities caused by the rigid vessel wall at the outlet region.

The lymphatic vessel contains a series of bi-leaflet valves that are modeled as visco-elastic solid structures anchored at the vessel wall (33). The opening and closing of the valves are passive, entirely controlled by forces imparted by the flowing fluid. In the absence of fluid flow, the valves are biased in the open position, consistent with experimental observations (46).

In simulations of longer vessels (31 lymphangions), the resistance is correspondingly larger, so we needed to increase the potential amplitude of the contractions by decreasing the minimum systolic diameter in the simulations. The contraction force is also increased by a factor of 4.5 to compensate for the larger resistance (see Suppplementary Material, increased Ca++ rate constant K_M_).

### Calcium fluxes, nitric oxide, and contraction force

Wall-generated forces simulate lymphatic muscle contractions, and are implemented using a simplified scheme for calcium dynamics in the muscle cells (32). Briefly, contractions are initiated when the Ca^++^ level exceeds a threshold, and the resultant contraction force is proportional to the Ca^++^ level. Ca^++^ accumulates in the cytoplasm of the muscle cells due to a constant leakage rate, which is enhanced by vessel stretch [which simulates stretch-sensitive ion channels (47, 48)]. Ca^++^ is depleted from the cytoplasm according to a recharge rate, which is increased by NO. NO production rate is proportional to the shear stress at the vessel wall (49, 50) and it degrades exponentially with a half-life of 0.31 s. Ca^++^ diffusion is restricted to the wall of the vessel (simulating gap junction transport), while NO diffuses and convects freely in the domain.

### Implementation

In practice, the vessel in Fig. 1 is divided into multiple 465 × 66 lattice sections for distributed GPU parallelization. Each vessel wall is discretized into 232 segments, and each valve leaflet is represented by 28 segments. Parameter values were either obtained from literature or estimated to produce vessel dynamics consistent with experimental observations (31–33, 51–53). The code is written in C++, and executed on a workstation with eight GPUs. The GPUs all are NVIDIA Quadro GP100, each with 3,584 CUDA cores. The workstation has two E5-2620 CPUs. We use CUDA to parallelize the computations on the GPUs. Each GPU calculates one or more regions. MPI (message passing interface) is used to exchange border data between neighboring regions. The lymphatic vessel model is only limited by computational power, and can be extended to simulate longer lymphatic vessels. For example, with eight GPUs, we can assign two computational domain units to each GPU, thus providing for 31 lymphangions (and 32 valves).

Note that no model parameters are changed when simulating the different gravitational forces (Supplementary Table S1). The observed changes in contractions naturally emerge due the mechanobiological mechanisms driven by NO and Ca^++^.

To initiate the simulations, we start with a high Ca^++^ concentration (just below the threshold level for contraction) at every vessel node. This is sufficient to induce rhythmic contractions, except in the cases with high adverse gravity. In these cases, an additional perturbation in the form of transiently increased Ca^++^ force constant is needed (Supplementary Fig. S1). Additional details of the mathematical model are included in the supplementary material.

## Results

**Fig. 2.**
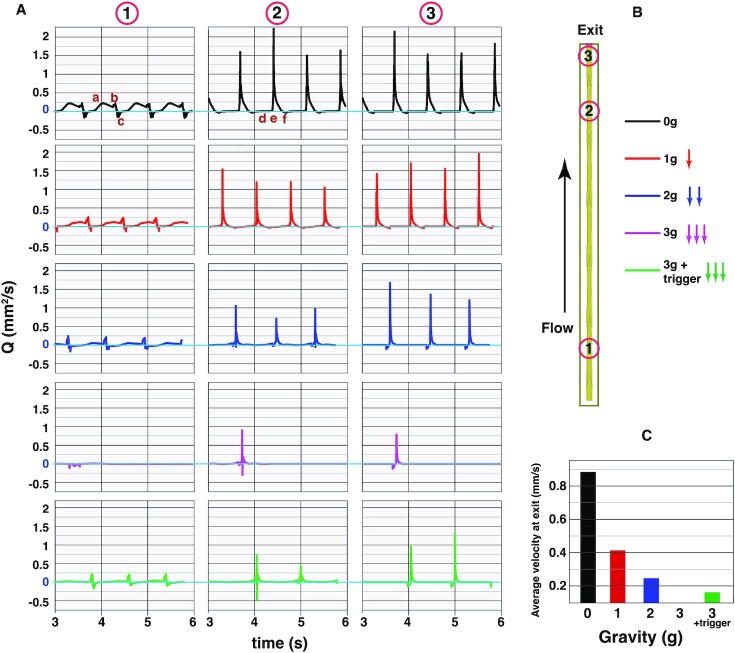
Gravitational forces affect lymphatic contractions and flow. (A) Flow rates as a function of time for three positions along the vessel and different gravitational forces (indicated in B). The time-averaged fluid velocities at the vessel outlet are plotted in (C). Simulations with 0, 1, 2, and  3g are shown.  "3g + trigger" indicates that we applied a pressure pulse to start the stalled contractions.

Because our simulated domain is relatively short, the effects of gravity are most easily studied by increasing the gravitational force artificially. To investigate how gravity affects lymphatic contractions and transport through lymphatic vessels, we simulated tissue drainage under various gravitational fields in a vertically oriented vessel, ranging from zero to three times normal gravity (g = 9.8 m/s^2^; Fig. 2). At the entrance region (1), the peak flow rates are lower, but more sustained compared with the short, high peaks seen in the downstream segments (2) and (3).

In the case of  0g, near the inlet region, positive flow is initiated as the vessel relaxes to the rest position after the previous contraction (Fig.   2, location 1,“a”). During this diastole phase, the upstream valve opens and fluid is pulled into the lymphangion. Note that the contractions are naturally synchronized, so downstream segments are also opening at the same time, increasing the volumetric flow rate at the entrance. The vessel contraction causes a shorter spike in outflow (Fig.   2, location 1, “b”). As diastole starts again, a small amount of retrograde flow occurs, which closes the downstream valve (Fig. 2, location 1, “c”), and the cycle repeats (see Supplementary Videos S1 and S2). In the downstream segments (Fig. 2, locations 2 and 3), the flow peaks are quite different. During diastole, the outlet vessel is closed and the net flow is very small (Fig. 2, location 2, “d”). This is because fluid is being pulled from upstream, where the flow rate is high, while there is no flux at the end of the vessel. Note that the fluid velocity in an expanding tube with a closed end depends on the tube length, wall velocity, and x-location along the tube. During contraction, the inlet valve closes, the outlet valves open, and the flow rate spikes, again due to the combined effects of multiple contracting lymphangions (Fig.   2, location 2, “e”). At the start of diastole, there is a small amount of backflow and the downstream valve closes (Fig. 2, location 2, “f”). Similar behavior is seen when gravity is introduced, but the flow rates are reduced (Fig. 2, 1 and 2 g; see Supplementary Videos S3 and S4).

The exit flow rate (averaged over the simulation) decreases as gravity increases (Fig. 2C). The hydrostatic pressure that a given lymphangion experiences is dependent on the length of the vessel, limb orientation, and the efficiency of the intraluminal valves. Interestingly, when gravity is increased to  3g, the additional pressure within the vessel lumen prevents the distal valve from opening, and the contractions stall: the cycle of calcium fluxes, flow, and NO production is disrupted, and flow stops (see Figs. 2, “ 3g”). In this condition, the vessel cannot generate enough force to open the valve(s). In addition, when gravity opposes the flow, the gradient of velocity $\partial {{\rm{v}}}_l/\partial {x}_n\ $ decreases. There is more resistance to flow due to the increased hydrostatic pressure, so each contraction moves less fluid. The decreased shear stress reduces the production of NO. In the model, NO enhances the release of calcium from myosin light chains and the depletion of cytoplasmic Ca^++^. With flow against gravity, less NO is available, thus delaying or preventing sufficient cytosolic calcium decay and extending the contraction cycle. Although similar “stalling” of contractions is often observed in vivo (11, 54, 55), a mechanistic explanation remains elusive.

**Fig. 3.**
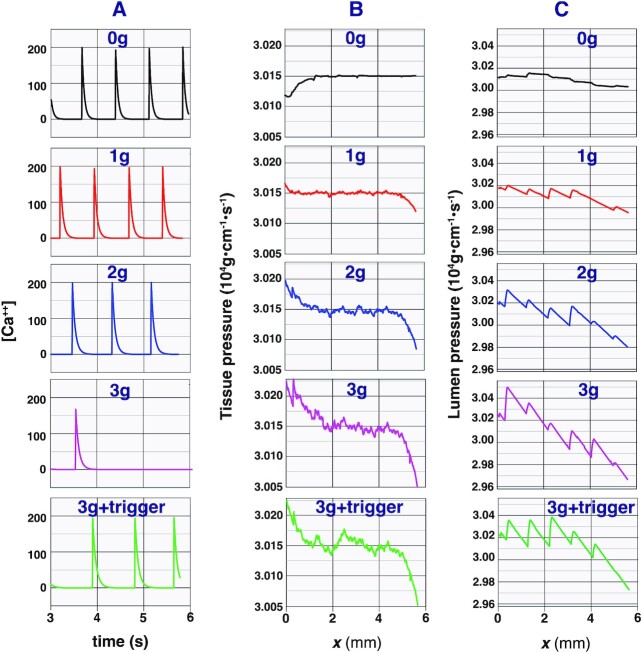
Calcium dynamics near the center of the vessel and fluid pressures in the domain. (A) Changes in Ca^++^ concentration in the middle segment over time. (B) Pressure in the tissue outside the vessel, averaged perpendicular to the vessel wall. (C) Average lumen pressure along the vessel. Simulations with 0, 1, 2, and 3 g are shown.  3g + trigger indicates that we applied a pressure pulse to start the stalled contractions.

When spontaneous contractions are prevented by gravitational forces, they can be initiated by simulating physical massage of the vessel (Figs. 2 and 3, “3 g + trigger”; Supplementary Fig. S1). In the simulations, we do this by transiently increasing the lymphatic muscle force, applied either in the initial segment (imposed for 0.133 s) or as a single traveling wave that propagates from entrance to outlet along the vessel wall. The lymphangion contractions are driven by calcium fluxes in the lymphatic muscle cells (Fig. 1), and the sustained cycle is facilitated by shear-induced NO produced in the endothelium. Transiently increasing the wall strength locally partially empties the vessel, reducing lumen pressure and creating additional NO, allowing another cycle to initiate. Under adverse gravitational forces (3 g in this example), this transient increase in lymphatic muscle force is sufficient to trigger stable oscillations. Clinically, lymph flow is often stimulated by tissue massage, and it has been shown that tissue/muscle movement can facilitate lymphatic transport, not by directly contracting lymphatic vessels, but by activating endogenous lymphatic contractility (11). The model suggests that the external perturbations function to decrease lumen pressure transiently while creating shear stress and producing NO. The transient increase in lymphatic muscle force may also create strain in the vessel wall to initiate the calcium action potentials through mechanically sensitive channels, thus “jump starting” the contractions. Further investigation is needed to determine the physiological mechanisms by which these external perturbations affect lymph transport.

The simulations also allow analysis of the fluid pressure in the surrounding tissue, which is an indication of edema (Fig. 3). In the absence of gravity (or when the vessel is horizontal), vessel contractions move fluid, and the pressure in the tissue near the entrance segment is the lowest (Fig. 3,  0g). When pumping against gravity, fluid pressure near the entrance region increases as gravity increases. When the gravity is increased to  3g, the vessel stops pumping, and the upstream tissue fluid pressure is greatest. Starting the contractions with a pressure pulse in this case can decrease tissue fluid pressure in the entrance region. Note that the tissue pressures increase or decrease primarily near the inlet and outlet and are relatively constant in the central tissue region. This is because our domain is finite and pressure differences are enhanced at the top and bottom of the “container.” The flattened pressure profile near the center is due to fluid entering and exiting the domain laterally, which buffers the pressure gradients and may simulate the drainage of fluid by other lymphatics outside the domain. Examining the average lumen pressures, we see the expected discontinuities at the valves. Because of the steep gradients in pressure along each lymphangion (relative to the surrounding tissue), the transwall pressures are not uniform along a given lymphangion segment, with higher gradients near the valves. This may contribute to the formation of “bulb” structures at the locations of the valves.

**Fig. 4.**
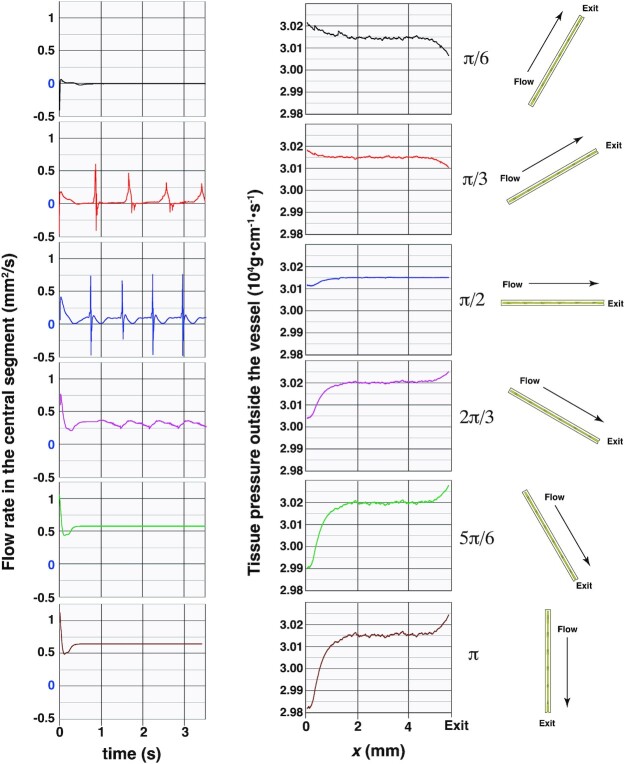
Flow rates and tissue pressures are affected by limb position. (A) Fluid flow in the central segment over time as a function of limb angle relative to gravity. (B) Pressure in the tissue outside the vessel, averaged perpendicular to the vessel segment for various limb angles. The magnitude of gravity is  3g, and we vary the angle relative to the vertical orientation. For the simulations of π/6 and π/3, an additional trigger (transiently increased Ca ^++^ force constant) was needed to initiate the contractions.

We next examined the effect of limb position (angle) on contractions and drainage. Keeping the gravity at  3g, we varied the angle of the vessel from zero (flow oriented against gravity) to π (flow oriented with gravity), and plotted the flux in the middle segment over time (Fig.   4). The simulations of the two steepest angles (α = π/6 and π/3) experience stalling similar to that seen with the vertical simulations (against gravity). As the direction of flow starts to align with the direction of gravity (i.e. the limb is elevated; α = 5π/6 and π), gravity drives the flow, generating steady NO and inhibiting the calcium fluxes. In these cases, the contractions stop, but flow still occurs, driven by gravity.

Next, we examined the effect of gravitational forces on valve opening and closing. This required simulating a longer vessel (31 lymphangions) to observe patterns in the valve dynamics. For longer vessels, the flow resistance and effects of gravity increase, and the contractions do not initiate as easily as with the shorter vessel. As with the shorter vessel exposed to high gravitational force, initiation of the contractions requires an external perturbation. After the initiation trigger, the vessel can pump without intervention. This “jump-start” procedure may simulate the initiation of lymphatic contractions by skeletomuscular motion (11, 56).

**Fig. 5.**
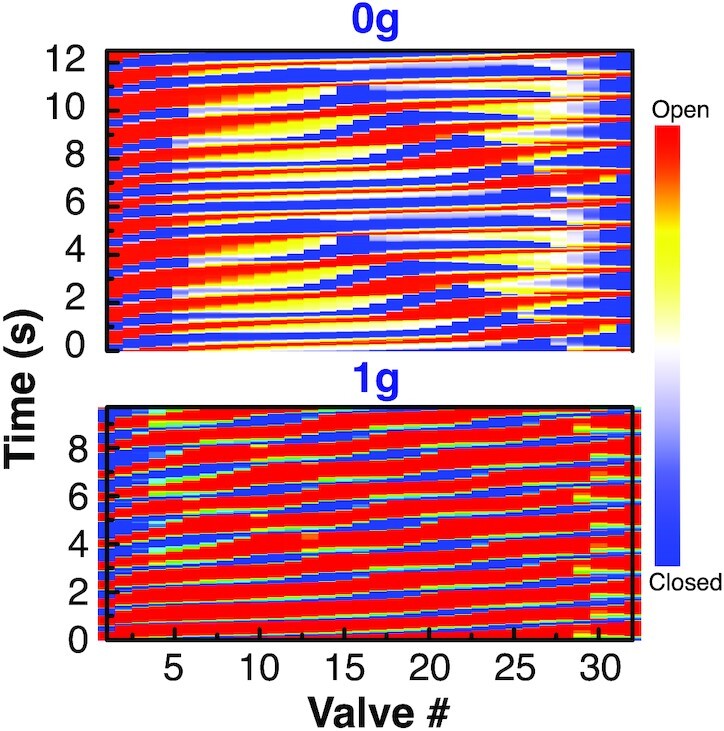
Valve dynamics in a vessel with 32 valves (31 lymphangions). Cases for 0 and 1 g adverse gravity are shown. As time proceeds from bottom to top, color changes indicate valve status. Red indicates fully open, and blue is closed.

To visualize the dynamics of the contractions and valves, we create color maps that show the valve status as a function of time and position along the vessel (blue and red colors indicate closed and open valves, respectively; Fig. 5). In these plots, we can follow a vertical line from bottom to top to see a single valve opening and closing (changing to red, then blue). So for example with 0 g, at *t* = 0, the first (leftmost) valve is blue, indicating that the valve at the inlet is closed. The contraction is starting from the inlet and propagating toward the outlet, as indicated by the progression from bottom left to top right in the red and blue color regions. The red color moves from left to right as time increases, indicating that the valves open sequentially from the inlet to the outlet. A vertical line near the left side of the plot most often intersects red color, so the valves near the inlet spend more time in the open position than the closed position, consistent with the analysis in Fig. 2. The initiation of the contractions can be inferred from this leftmost valve as well: when a contraction is initiated, this valve will close, changing the color from red to blue.

In contrast to the left side of the plot (vessel entrance), a vertical line near the right side (vessel outlet) has more blue color than red, so valves in this region are mostly closed. This difference is driven by the difference in fluid velocities during contraction and relaxation. As the vessel contracts, fluid is rapidly pushed through the exit, resulting in short opening times for valves at the outlet. On the other hand, vessel relaxation back to baseline is slower, so pulling fluid from the inlet requires the valves in that region to stay open longer. In addition, there is more flow resistance downstream from the first lymphangions, and their contractions are more prolonged, as also demonstrated in Fig. 2. This contributes to the prolonged valve opening.

We can also examine horizontal lines to see how many valves are open at a given time. As a horizontal line moves up through the plot (advances in time), we see that the line often intersects with long stretches of red color, indicating that multiple valves in the sequence are all open at the same time. In general, we see that ∼20 sequential valves near the central region are all open or closed at a given time step, corresponding to a distance of ∼2.23 cm.

Comparing 0g with  1g adverse gravitational force, there are notable differences in valve dynamics. First, there is more frequent red color with  1g, indicating that there are in general, more open valves when gravity is opposing flow. This seemingly counterintuitive result makes sense when considering the flow resistance argument discussed earlier. Against gravity, there is more resistance to flow, and the vessel wall velocity during systole is slower. This prolongs the contraction, keeping the downstream valves open longer. The contractions against gravity are also more synchronized and tightly packed. In general, the distance between closed valves is reduced to the range of 10 to 12 lymphangions. This indicates that as opposed to the  0g case, where long stretches of vessel contract nearly simultaneously, when pumping against gravity, the lymphangions contract more sequentially, in “bucket brigade” style.

## Discussion

The lymphatic system is a complex, self-regulated system that functions to maintaining fluid homeostasis. One clinical manifestation of impaired lymphatic function is lymphedema. There is abundant evidence that gravitational forces and limb position affect fluid homeostasis and lymphatic function, but detailed mechanistic analyses are lacking. To address this issue, we developed a computational model that includes explicit fluid dynamics and known mechanobiological mechanisms of lymphatic contractions.

The model has several notable limitations. Due to computational limitations, our simulations are limited to a 2D channel, and as such cannot fully represent the 3D structure of the valves or the circular cross section of a real vessel. As a result, the flow rates reported in Figs. 2 and 4 are presented in units of area per unit time rather than volume per unit time as would be expected from a 3D model. A volumetric flow rate might be estimated by multiplying by the width of vessel in the direction normal to the plane of the simulation, but this approach should not be expected to exactly match physiological flow rates. For example, it is well known that steady 2D flow between parallel plates has a different mean to peak velocity ratio than found in a 3D circular tube of the same diameter. Nonetheless, our comparisons among 2D simulations should be expected to be internally consistent, and the mechanobiological feedback mechanisms should be consistent in 3D. In addition, our domain is, by necessity, limited, and boundary effects influence the pressures predicted in Figs. 3 and 4. However, the general trends are intuitively correct, and the changes in contraction behavior in response to perturbations agree well with those observed in animal models, ex vivo preparations and clinical observations.

Our simulations of self-sustaining contractions of long vessels show that the interval between closed valves may be many lymphangions in length, indicating that fluid and chemical signals can induce coordinated action of a chain lymphangions, even in the absence of external pacemaking. The simulations suggest that gravitational forces may exacerbate lymphedema by increasing distal pressures and altering tissue and luminal pressure gradients, ejection velocities, and wall shear stresses in the lymphatic vessels. This, in turn, changes the dynamics of NO production and calcium-induced contractions. Gravitational forces also alter the propagation of the lymphatic contractions along the vessel wall, so that the velocity is smaller, and the coordination of valve opening and closing is restricted to shorter sequences of lymphangions. This is apparently necessary to protect the distal vessels from higher pressure, but makes the pumping less productive.

The simulations also show that gravitational forces can prevent the distal valves from opening, preventing synchronized contractions. In these cases (seen primarily in the simulations with 3 g), our usual startup procedure—which involves seeding a high level of Ca^++^ along the vessel wall—leads to a single contraction that dies out due to a lack of shear-induced NO and insufficient force to open valves. Subsequently, the fluid and vessel wall dynamics relax, and flow stops (see Supplementary Fig. S1). In these simulations, a stronger perturbation is needed, and the contractions can be restarted by simulating exogenous mechanical perturbations (similar to skeletal muscle motion or massage). This can be accomplished by briefly enhancing the force produced by the lymphatic muscle cells to induce wall motion, either localized at the initial segment or imposed as a traveling wave down the vessel. This has the effects of transiently decreasing lumen pressure as the vessel empties and increasing shear stress and NO, which can “reset” the contraction cycle.

In summary, our results highlight the importance of gravitational forces, limb position, and external perturbations on lymphatic vessel function. Future experimental studies may shed more light on the mechanisms by which mechanical perturbations can influence lymph transport.

## Authors' Contributions

J.W.B., T.P.P., and L.L.M. designed research; H.L. and H.W. performed research; H.L. and H.W. developed mathematical model and performed numerical simulations; H.L., J.W.B., T.P.P., and L.L.M. analyzed data; and H.L., J.W.B., T.P.P., and L.L.M. wrote the paper.

## Supplementary Material

pgac237_Supplemental_Files

## Data Availability

The code used in this study has been made available at https://github.com/llm0/Autonomous-lymphatic-contractions.
