# Supplementary material for: Computational simulations of the effects of gravity on lymphatic transport
Source: PNAS Nexus. 2022 Oct 18;1(5):pgac237. doi: 10.1093/pnasnexus/pgac237 (PMC9802413; doi:10.1093/pnasnexus/pgac237)
Supplement: pgac237_Supplemental_Files [file pgac237_supplemental_files.zip › PNASNEXUS-PNASNEXUS-2022-00269-T-s05.pdf]

# Supplementary Information for

## Computational simulations of the effects of gravity on lymphatic transport

**Huabing Li, Huajian Wei, Timothy P. Padera, James W. Baish and Lance Munn.**

**Lance Munn.**
**E-mail: munn@steele.mgh.harvard.edu**

### This PDF file includes:

Figs. S1 to S2 (not allowed for Brief Reports)
Table S1 (not allowed for Brief Reports)
Legends for Movies S1 to S4
SI References

### Other supplementary materials for this manuscript include the following:

Movies S1 to S4

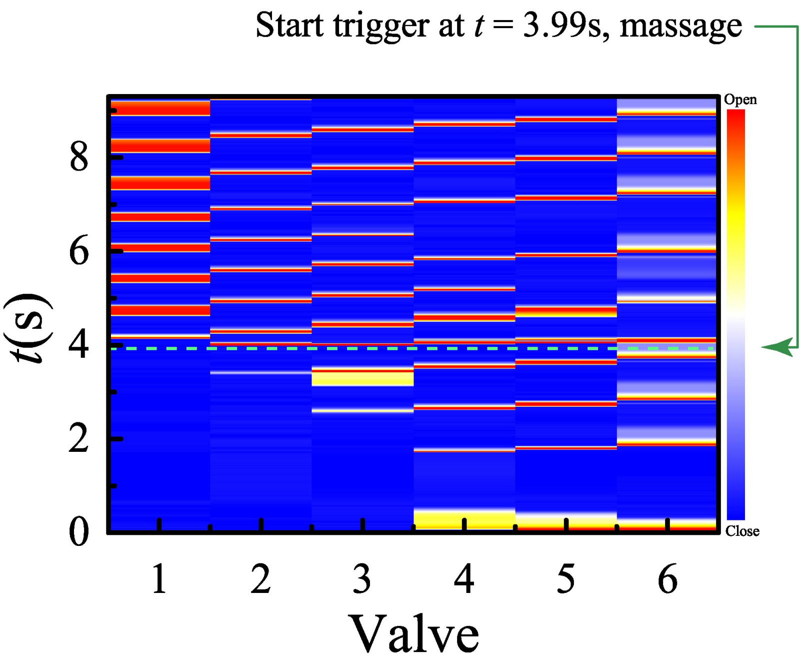


**Fig. S1.** Restarting stalled pumping. With large adverse gravitational force (3g), the normal startup procedure, which involves setting the initial $\mathrm{Ca}^{2+}$ at the wall to a high, sub-threshold value, does not result in sustained contractions. Instead, the valves quickly close along the length of the vessel (blue color, 0-2s). Downstream valves transiently open and close for a few seconds as the system relaxes, and the visco-elastic vessel wall passively stretches and contracts in response to the fluid forces (2-4s). At 3.99s, we apply a transient increase in the force constant that controls the $\mathrm{Ca}^{2+}$ force, propagated as a single wave down the vessel. This is sufficient to create sustained, synchronized pumping (4-9s).

## Methods

**Vessel wall and the valve.** Both the wall and the valve are discretized into segments, which can only move along the y (vertical) direction. They both have bending force:

$$F_B = -K_B((y - y_m) + (y - y_n)), \quad [1]$$

where $y_m$ and $y_n$ are the $y$-positions of the neighboring segments. For the vessel $K_B$ is constant, but for the valve, it's stronger for the anchor point and softer for the tip (1); thus we assume the bending rigidity for the valves as:

$$K_B^v = \frac{2(K_0^v - K_R^v)}{1 + \exp(Ai/n)} + K_R^v, n \geq i \geq 0, \quad [2]$$

where $i$ indicates segment number and $n$ is the total number of valve segments. $K_0^v$ is the maximum of $K_B^v$ at anchor point. $K_R^v$ is an approximation of the minimum of $K_B^v$, and the coefficient $A$ adjusts the strength of stiffness variation along the valve. The elastic force from the tissue is

$$F_E = -K_E(R - R_0), \quad [3]$$

where $R$ is the radius of the vessel or valve, $R_0$ is the rest radius. For the vessel, $R_0$ is a constant, but for the valve, to maintain the correct curvature seen in experiments, we impose a piecewise linear shape at rest:

$$y_0 = y_{l0} \pm \sqrt{(x - x_0)/B}, \quad [4]$$

where $y_{l0}$ is the rest position of the vessel, $x_0$ is the anchor point of the valve. '-' and '+' indicate upper and lower valve leaflets. $B$ can adjust the rest position so that it is biased to stay open or close. We specify the minimum vessel radius $R_l$ to limit the extent of the contractions. The valve also has a limit position to avoid excessive opening, specified by $B$. The structures are visco-elastic, so the viscous resistance force is introduced as:

$$F_r = -K_r v. \quad [5]$$

The minus sign means that $F_r$ always acts in the direction opposing wall velocity $v$.

There are lymphatic muscle cells (LMCs) on the lymphatic vessel, which can contract as calcium accumulates in the cytoplasm of the LMCs. The effect of calcium is known to be tempered by nitric oxide. The lymphatic muscle force depends on the concentrations of Ca and nitric oxide according to (2):

 

$$F_M = K_M(\frac{C_{\mathrm{Ca}}}{1+C_{\mathrm{Ca}}})(\frac{2R}{R+R_{\mathrm{Ca}}})(\frac{1}{1+K_{\mathrm{NO}}C_{\mathrm{NO}}}), \quad [6]$$

where $C_{\mathrm{Ca}}$ and $C_{\mathrm{NO}}$ are the concentration of calcium and NO respectively, and $K_M$ is the coefficient determining the strength of action. Ca production and the dynamics of Ca in the vessel wall (which includes diffusion) can be described as (2–7):

$$\begin{aligned}\triangle C_{\mathrm{Ca}}(\mathbf{x},t) = \quad & D_{\mathrm{Ca}}\nabla^2 C_{\mathrm{Ca}}(\mathbf{x},t)\triangle t \\ & +(-K^{-}_{\mathrm{Ca}}(1+K_{\mathrm{Ca,NO}}C_{\mathrm{NO}})C_{\mathrm{Ca}}+K^{+}_{\mathrm{Ca}} \\ & +K^{+}_{\mathrm{Ca}}(\frac{(R-R_l)}{(R_{\mathrm{Ca}}-R_l)})^{11} \\ & +10K^{+}_{\delta}\delta\uparrow(C_{\mathrm{th}},C_{\mathrm{Ca}}))\lambda\triangle t. \end{aligned} \quad [7]$$

where the last term simulates calcium-induced calcium spikes.

Production and diffusion of nitric oxide can be simulated through:

$$\begin{aligned}\triangle C_{\mathrm{NO}}(\mathbf{x},t) \quad & = D_{\mathrm{NO}}\nabla^2 C_{\mathrm{NO}}(\mathbf{x},t)\triangle t - \mathbf{u}\cdot\nabla C_{\mathrm{NO}}(\mathbf{x},t)\triangle t \\ & +(-K^{-}_{\mathrm{NO}}C_{\mathrm{NO}}(\mathbf{x},t)+K^{+}_{\mathrm{NO}}\mid\frac{\partial v_l}{\partial x_n}\mid)\lambda\triangle t, \end{aligned} \quad [8]$$

where $C_{\mathrm{NO}}$ is the concentration of NO. $D_{\mathrm{NO}}$ is the diffusion coefficient of NO. The third term represents production due to wall shear stress. Each segment moves according to the Newtonian law calculated by a so-called half-step 'leap-frog' scheme (8).

**Extreme treatment.** "Extreme" treatment is used when one body approaches closely to a limit position or two bodies are too close to each other. As shown in Fig. S2 (A), if a segment is too close to a limit position (for example, the vessel contracts too much and the gap between the upper wall and the limit position $R - R_l = \delta < \Delta$, we multiply $F_E$ by $(\frac{\Delta}{\delta})^{11}$. This transient but strongly increasing force prevents further approach and avoids numerical instabilities. The valve also has a limit position described by Eq. (4) where $B$ is the maximum. If a valve is closing and the two leaflets are too close together, we also apply a lubrication force (9) to stabilize the membranes of the valve when there is no fluid node between them. In our simulation, the Newtonian time of the vessel and valve is 1/100 of lattice Boltzmann time step.

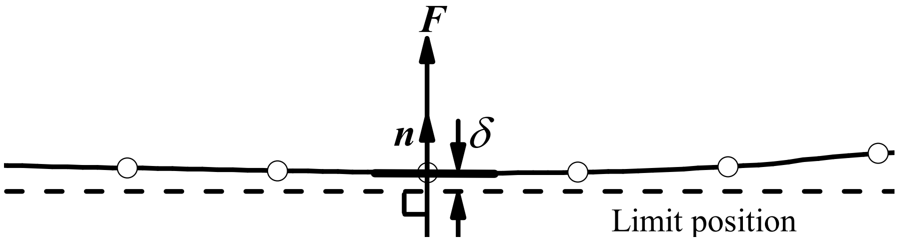


**Fig. S2.** Schematic diagram of a membrane approaching too close to a limit position. $\delta$ is the gap between a membrane and the limit position. $\boldsymbol{F}$ is a rapidly increasing force as $\delta$.

## Simulation parameters

The diameter of the vessel at rest is assumed to be $D' = 0.01cm$. The lymph inside is treated as water with kinematic viscosity of $\nu' = 0.01cm^2/s$ and density of $\rho' = 1g/cm^3$. The density of valves is also treated as water, but the density of the vessel is eighty times that of water. In the simulation, we choose the vessel rest diameter as $D = 25$ lattice nodes, and the relaxation time $\tau = 0.75$; thus the kinematic viscosity $\nu = (2\tau - 1)/6 = 0.0833$, the density at the inlet is $\rho = 1$, the initial fluid density $\rho_0 = 1$, and the velocity is zero. The initial calcium concentration at each membrane segment is 0.0999, which is close but below the threshold of calcium. The initial NO concentration at each lattice is zero. Each time step is $T = \frac{\nu}{\nu'}(\frac{D'}{D})^2 = 1.33 \times 10^{-6}s$, each lattice length is $L = \frac{D'}{D} = 0.0004cm$, and the pressure unit is $P = (L/T)^2 \mathrm{g}\cdot\mathrm{cm}^{-3} = 9.045 \times 10^4 \mathrm{g}\cdot\mathrm{cm}^{-1}\cdot\mathrm{s}^{-2}$. Other parameters are given in table S1. Here, in order to use parallel computing to calculate multi-lymphangion contracting together using MPI, when we discretize the membranes of the vessel and valves, we set the length of each segment to two lattice units. First we only use three GPUs to calculate three lymphangions (each including two valves) contracting under different gravity. Then we calculate 16 lymphangions contracting.

| Parameter | Definition | Value | Units | Source |
|---|---|---|---|---|
| **Chemical properties of NO and** $Ca^{2+}$ | | | | |
| $D_{NO}$ | NO diffusivity | $1.2 \times 10^{-4}$ | $cm^2/s$ | $3.0 \times 10^{-5}(cm^2/s)$(10) |
| $K^-_{NO}$ | NO degradation rate constant | 75.1 | $s^{-1}$ | Estimated |
| $K^+_{NO}$ | NO production rate constant | 20 | Dimensionless | Estimated |
| $D_{Ca}$ | $Ca^{2+}$ diffusivity | $5.02 \times 10^{-7}$ | $cm^2/s$ | $6.5 \times 10^{-7} cm^2/s$ (11) |
| $K^-_{Ca}$ | $Ca^{2+}$ degradation rate constant | 375.9 | $s^{-1}$ | Estimated |
| $K^+_{Ca}$ | $Ca^{2+}$ production rate constant | 22.6 | $s^{-1}$ | Estimated |
| $K^+_{\delta}$ | $Ca^{2+}$ production rate constant | $1.1 \times 10^5$ | $s^{-1}$ | Estimated |
| $C_{th}$ | $Ca^{2+}$ threshold | 0.1 | Dimensionless | Estimated |
| $R_{Ca}$ | Threshold radius for $Ca^{2+}$ channel sensitization | $R_0 - 0.5$ | $L$ | Estimated |
| $K_{Ca,NO}$ | Rate constant for NO inhibition of $Ca^{2+}$ | 0.5 | Dimensionless | Estimated |
| $\lambda$ | Chemical reaction rate constant | 0.03 | Dimensionless | Estimated |
| **VESSEL** | | | | |
| $K_M$ | Force constant for $Ca^{2+}$ | $1.0 \times 10^{-4}$ | $g \cdot cm/s^2$ | Estimated |
| $K_E$ | Young elastic modulus of the vessel | 407.0 | $dynes/cm^2$ | Estimated |
| $K_B$ | Young bending modulus of the vessel | $3.6 \times 10^4$ | $dynes/cm^2$ | $10^6(dynes/cm^2)$(12) |
| $K_r$ | Viscosity coefficient of vessel | $4.8 \times 10^{-9}$ | $g/s$ | Estimated |
| $K_{NO}$ | NO inhibition of force | 0.3 | Dimensionless | Estimated |
| $R_l$ | Limit radius | 7.5 | $L$ | Estimated |
| $R_0$ | Rest radius of the vessel | 12.5 | $L$ | Estimated |
| **VALVE** | | | | |
| $A$ | How soft the valve is | 6 | Dimensionless | Estimated |
| $B$ | How much the valve biased to open | 1500 | $cm^{-1}$ | Estimated |
| $K^v_E$ | Young elastic modulus of valves | $9.0 \times 10^{-4}$ | $dynes/cm^2$ | Estimated |
| $K^v_0$ | Young Bending modulus of the base of valves | $7.2 \times 10^4$ | $dynes/cm^2$ | $10^6(dynes/cm^2)$ (12) |
| $K^v_R$ | Young bending modulus of the tip of valves | 0.018 | $dynes/cm^2$ | $0.1k^v_0$ (1) |
| $K^v_r$ | Viscosity coefficient of the valve membrane | $4.8 \times 10^{-9}$ | $g/s$ | Estimated |
| **VESSEL & VALVE** | | | | |
| $\triangle$ | | 0.5 | $L$ | Estimated |

**Table S1. Chemical parameters of NO and $Ca^{2+}$; Mechanical parameters of the fluid, vessel wall and valves.**

**Movie S1. Lymphatic vessel contractions without gravity. The color map shows the nitric oxide levels.**

**Movie S2. Lymphatic vessel contractions without gravity. The same simulations as S1, but showing pressure in the colormap.**

**Movie S3. Lymphatic vessel contractions with 1g opposing the flow. The color map shows the nitric oxide levels.**

**Movie S4. . Lymphatic vessel contractions with 1g opposing the flow. The same simulations as S3, but showing pressure in the colormap.**

## References


1. GA Buxton, N Clarke, Computational phlebology: the simulation of a vein valve. *J. biological physics* **32**, 507–521 (2006).
2. C Kunert, JW Baish, S Liao, TP Padera, LL Munn, Mechanobiological oscillators control lymph flow. *Proc. Natl. Acad. Sci.* **112**, 10938–10943 (2015).
3. Y Osipchuk, M Cahalan, Cell-to-cell spread of calcium signals mediated by atp receptors in mast cells. *Nature* **359**, 241 (1992).
4. EA Newman, KR Zahs, Calcium waves in retinal glial cells. *Science* **275**, 844–847 (1997).
5. AC Charles, JE Merrill, ER Dirksen, MJ Sandersont, Intercellular signaling in glial cells: calcium waves and oscillations in response to mechanical stimulation and glutamate. *Neuron* **6**, 983–992 (1991).
6. A Kapela, A Bezerianos, NM Tsoukias, A mathematical model of ca2+ dynamics in rat mesenteric smooth muscle cell: agonist and no stimulation. *J. theoretical biology* **253**, 238–260 (2008).
7. M Jafarnejad, et al., Measurement of shear stress-mediated intracellular calcium dynamics in human dermal lymphatic endothelial cells. *Am. J. Physiol. Circ. Physiol.* **308**, H697–H706 (2015).
8. MP Allen, DJ Tildesley, *Computer simulation of liquids.* (Oxford university press), (2017).
9. X Yuan, R Ball, Rheology of hydrodynamically interacting concentrated hard disks. *The J. chemical physics* **101**, 9016–9021 (1994).

10. IG Zacharia, WM Deen, Diffusivity and solubility of nitric oxide in water and saline. *Annals biomedical engineering* **33**, 214–222 (2005).
11. NL Allbritton, T Meyer, L Stryer, Range of messenger action of calcium ion and inositol 1, 4, 5-trisphosphate. *Science* **258**, 1812–1815 (1992).
12. R Wesly, RN Vaishnav, J Fuchs, DJ Patel, J Greenfield Jr, Static linear and nonlinear elastic properties of normal and arterialized venous tissue in dog and man. *Circ. research* **37**, 509–520 (1975).
